# Comparing multi-texture fibrosis analysis versus binary opacity-based abnormality detection for quantitative assessment of idiopathic pulmonary fibrosis

**DOI:** 10.1038/s41598-025-85135-7

**Published:** 2025-01-09

**Authors:** Sebastian Nowak, Dominik Creuzberg, Maike Theis, Carmen Pizarro, Alexander Isaak, Claus C. Pieper, Julian A. Luetkens, Dirk Skowasch, Alois M. Sprinkart, Daniel Kütting

**Affiliations:** 1 Department of Diagnostic and Interventional Radiology, University Hospital Bonn, Venusberg-Campus 1, 53127 Bonn, Germany; 2 Department of Internal Medicine II, Cardiology/Pneumology, University Hospital Bonn, Bonn, Germany

**Keywords:** Computed tomography, Idiopathic pulmonary fibrosis, Opacified lung, Spirometry, Machine learning, Survival analysis, Three-dimensional imaging, Respiratory tract diseases, Prognostic markers

## Abstract

Automated tools for quantification of idiopathic pulmonary fibrosis (IPF) can aid in ensuring reproducibility, however their complexity and costs can differ substantially. In this retrospective study, two automated tools were compared in 45 patients with biopsy proven (12/45) and imaging-based (33/45) IPF diagnosis (mean age 74 ± 9 years, 37 male) for quantification of pulmonary fibrosis in CT. First, a tool that identifies multiple characteristic lung texture features was applied to measure multi-texture fibrotic lung (MTFL) by combining the amount of ground glass, reticulation, and honeycombing. Opacity-based fibrotic lung (OFL) was measured by a second tool that performs a simpler binary classification of tissue into either normal or opacified lung and was originally developed for quantifying pneumonia. Differences in quantification of MTFL and OFL were assessed by Mann-Whitney U-test and Pearson correlation (r). Also, correlation with spirometry parameters (percent predicted total lung capacity (TLC), percent predicted vital capacity (VC), percent predicted forced expiratory volume in 1 s (FEV_1_), diffusing capacity of the lungs for carbon monoxide (DL_CO_), partial pressure of oxygen (P_O2_) and carbon dioxide (P_CO2_)) were assessed by r. The prognostic values for 3-year patient survival of OFL, LSS and MTFL were investigated by multivariable Cox-proportional-hazards (CPH) models including sex, age and TLC and including sex, age and VC. Also, Kaplan-Meier analysis with log rank test between subgroups separated by median OFL and MTFL were conducted. No significant difference between OFL and MTFL was observed (median and interquartile range: OFL = 29% [20-38%], MTFL = 31% [19-45%]; *P* = 0.44). For OFL significant correlation was observed to MTFL (*r* = 0.93, *P* < 0.01) and VC (*r*=-0.50, *P* = 0.03). For MTFL no significant correlation to spirometry parameters was found. The total time for one analysis was lower for the automated MTFL (MTFL: 313 ± 25s vs. OFL: 612 ± 61s, *P* < 0.001). Both analyses were significant predictors in the multivariable CPH analysis including TLC (hazard-ratios: MTFL 1.03 [1.01–1.06], *P* = 0.02; OFL 1.03 [1.00-1.06], *P* = 0.03). No parameter was a significant predictor in the CPH models including VC (hazard-ratios: MTFL 1.01 [0.98–1.04], *P* = 1; OFL 1.01 [0.97–1.05], *P* = 1). OFL showed significance in Kaplan-Meier analysis (MTFL: *P* = 0.17; OFL: *P* = 0.03). Using a simple opacity-based quantification of pulmonary fibrosis in IPF patients displayed similar results and prognostic value compared to a more complex multi-texture based analysis.

## Introduction

Idiopathic pulmonary fibrosis (IPF) is a rare and dangerous disease with high mortality^1^. IPF is characterized by irreversible fibrotic remodeling of the bronchial system without an identifiable cause, ultimately leading to insufficient gas exchange or heart failure^2,3^. In addition to pulmonary function tests and microscopic parenchymal assessment, CT imaging has an important role in the diagnosis and quantification of disease extent. Prior to physiological assessment and imaging, progressive exertional dyspnea is often misdiagnosed, which may lead to delayed diagnosis of IPF^4^. The greater extent of fibrotic tissue changes at the time of delayed diagnosis leads to a worse prognosis^5^.

Reliable quantification of fibrotic lung alterations by radiological imaging is increasingly demanded for the diagnosis and monitoring of the progression of pulmonary fibrosis, especially in IPF^6,7^. However, visual quantifications of fibrotic lung disease are subjective, which can lead to inter-reader differences. Furthermore, assessment requires expertise in pulmonary imaging, however the availability of qualified radiologists/pulmonologists may be limited in some regions^7–9^. The recently introduced subtype of progressive fibrosis in interstitial lung (PF-ILD) is defined by the presence of at least 10% fibrosis that shows signs of progressive fibrotic alterations and worsening of clinical symptoms^8^. Inter-reader differences in visual assessment of pulmonary fibrosis can therefore lead to misinterpretations that influence therapy decisions. Automated objective pulmonary fibrosis quantification tools that ensure general reproducibility and traceability of results, are cost-effective and widely available, are therefore of high interest.

Recent studies suggest that automated software-based quantifications offer benefits over purely visual assessment of fibrotic alterations in diffuse lung disease^7,10–12^. In principle, CT pulmonary parenchymal analysis tools can vary in complexity ranging from simple binary discrimination of lung tissue in normal or pathological up to sophisticated analyses of parenchymal changes by distinguishing multiple characteristic lung texture features (i.e. ground glass, reticulation, honeycombing). As most of these changes found in pulmonary fibrosis alter pulmonary opacity, these sophisticated analyses might not be necessary compared to a simple opacity-based, binary differentiation of healthy and diseased lung parenchyma. Thus, it was the aim of this study to compare the diagnostic and prognostic value of a simpler opacity-based quantification of pulmonary fibrosis to a more sophisticated multi-texture lung analysis algorithm.

## Methods

**Fig. 1:**
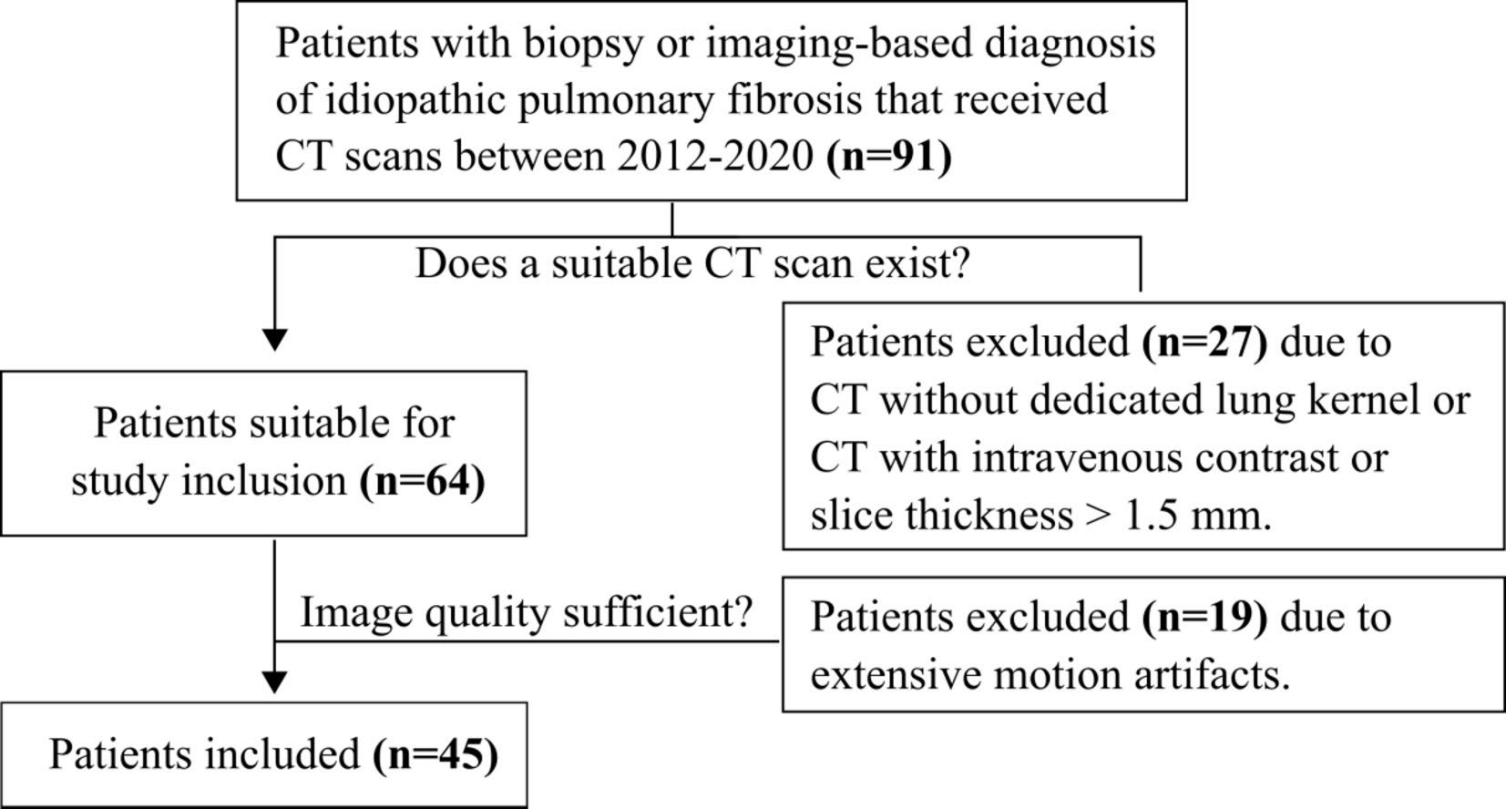
Cohort selection flow chart. The retrospective study included patients with biopsy or imaging-based diagnosis of idiopathic pulmonary fibrosis who received high-resolution and non-contrast CT scans.

This retrospective study was performed in accordance with the Declaration of Helsinki and approved by the institutional review board with a waiver of written informed consent. Patients with biopsy or imaging-based diagnosis of idiopathic pulmonary fibrosis that received CT scans between 2012 and 2020 were included. Patients were excluded from this study, if CT scans were not available in a dedicated lung kernel, if slice thickness was > 1.5 mm, if scans were acquired with intravenous contrast and if extensive motion artifacts were present in the respective scans. All patients with image-based diagnosis presented a usual interstitial pneumonia pattern and were otherwise unremarkable in interstitial lung disease workup^13^. Details regarding patient selection are listed in Fig. 1.

Quantifications for assessing pulmonary fibrosis were performed using two software tools:

1. Opacity based quantification of fibrosis: For determining OFL, a CT-based pneumonia analysis tool was used, which identifies voxels with opacified lung (including reticulation, ground glass and consolidation) in % for the entire lung^14^. Additionally, severity of pulmonary affection is estimated with a lung severity score (LSS) that assesses local severity for each lobe based on the percentage of affected pulmonary parenchyma (0: <1%, 1: ≤25%, 2: ≤50%, 3: ≤75%). LSS is calculated by accumulation of all local scores^15^. The tool is currently a research only prototype and included in the SyngoVia software (Siemens Healthineers).
2. Multi-texture based quantification of fibrosis: For calculation of MTFL a dedicated lung-texture analysis tool was applied that identifies a variety of sub-features of fibrotic alteration that are typical or at least predominant in patients with IPF^16^. MTFL was defined as the combined amount of ground glass, honeycombing and reticular abnormalities. The computer-aided lung informatics for pathology evaluation and ratings (CALIPER) tool is part of the Imbio lung texture analysis software (Imbio) and was created by the Biomedical Imaging Resource Laboratory of the Mayo Clinic, Minnesota.

Additionally, duration of data extraction necessary for analysis, image analysis and manual corrections of analysis (if possible) were assessed for the two tools.

### Statistical analysis

Estimation of lung fibrosis by OFL and LSS by the Pneumonia tool required some manual interactions. Analyses were performed by two radiologists with 11 (D.K.) and 5 years (D.C.) of experience in chest imaging. To assess inter-reader reproducibility intra-class correlation coefficient (ICC) with 95% confidence intervals (CI) was determined. Estimation of lung fibrosis by MTFL was a fully automated process without option of manual corrections and was conducted by (D.K.). Datasets were anonymized and randomly presented to the readers preventing direct correlation between the two analysis tools. To further avoid bias, assessment of OFL was performed first and subsequently, after an interval of 2 weeks, MTFL was determined.

Statistical data analysis was performed in Python v3.9.12. Correlation and mean differences between OFL and MTFL were assessed with linear regression analysis with Pearson correlation (r) and Bland-Altman analysis using Scikit-learn v1.1.2 and SciPy v1.9.0^17,18^. Differences in OFL and MTFL were assessed with Mann-Whitney U-test. Also, regression analysis was used to investigate correlation between OFL, LSS and MTFL with spirometry parameters for the subset of patients for whom these information were available. These parameters were percent predicted total lung capacity (TLC), percent predicted vital capacity (VC), percent predicted forced expiratory volume in 1 s (FEV_1_), diffusing capacity of the lungs for carbon monoxide (DL_CO_), partial pressure of oxygen (P_O2_) and carbon dioxide (P_CO2_). Correction for multiple comparison of 19 regression analysis was conducted by Bonferroni method. Statistical significance was defined as P-value < 0.05.

The prognostic value of OFL, LSS and MTFL for 3-year survival was investigated in multivariable Cox-proportional-hazards (CPH) models together with basic clinical attributes (sex, age) and with TLC, and separately with sex, age and VC. We aimed for a low number of variables in CPH analysis to mitigate effects of overfitting. Correction for multiple comparison in three CPH models was also conducted by Bonferroni method. CPH analysis was performed in SPSS Statistics v27.0. Additionally, Kaplan-Meier analysis was conducted for OFL, LSS and MTFL with log rank test between patient groups separated by median using lifelines v0.28.0^19^.

## Results

**Fig. 2:**
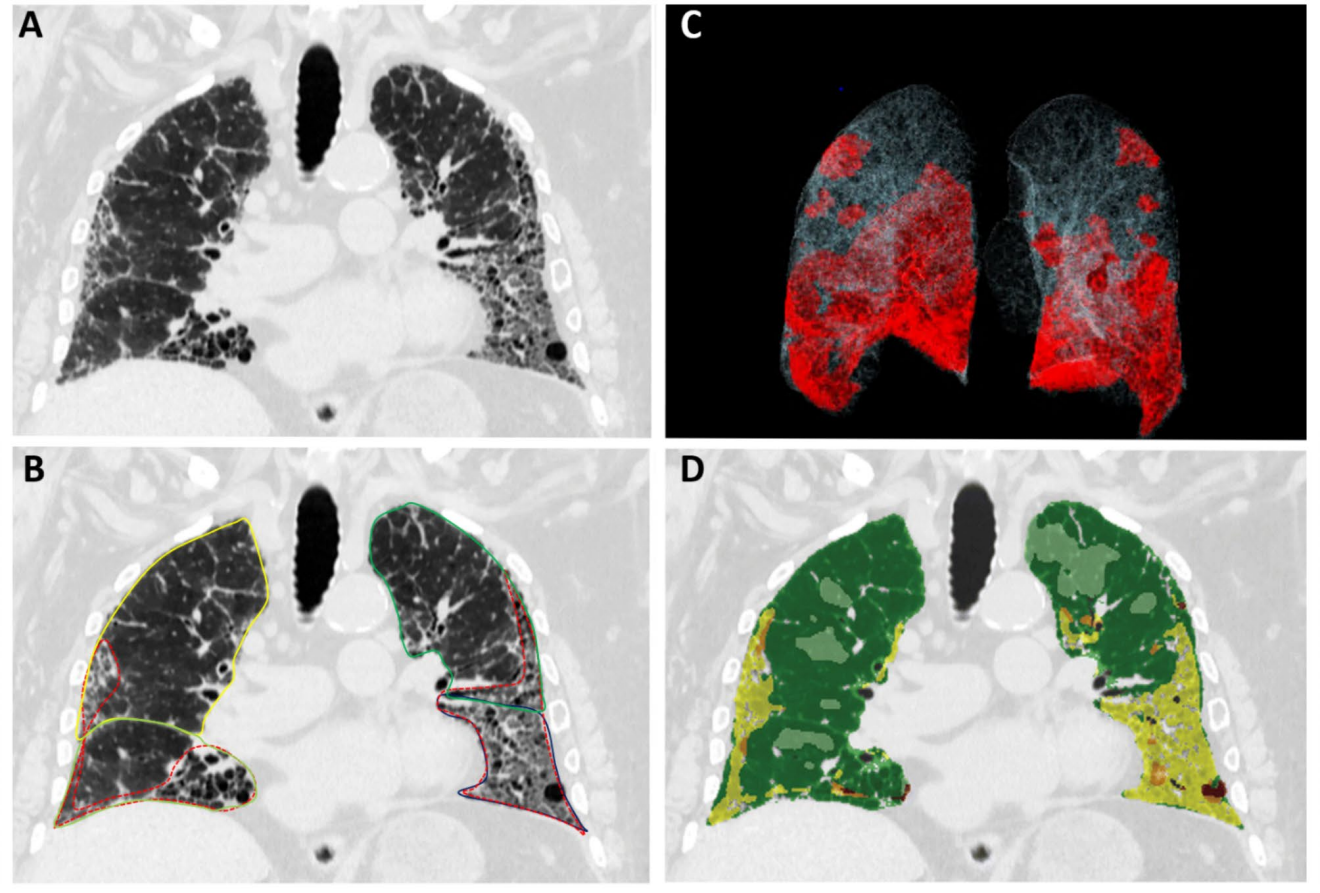
Exemplary results of the multi-texture lung analysis tool and the opacity-based lung analysis tool. CT in coronal view of a 62 year old patient with idiopathic pulmonary fibrosis (**A**), analyzed by the opacity-based Pneumonia tool (Siemens Healthineers), the red lines represent the segmented fibrotic areas (**B**) with additional 3D reconstruction (**C**). (**D**) Analysis with the multi-texture lung analysis tool (CALIPER, Biomedical imaging resource laboratory, Mayo Clinic) with the yellow areas representing fibrotic altered tissue.

A total of 45 patients (mean age 74 ± 9 years, 37 male) with biopsy (12/45) or imaging-based (33/45) proven IPF were included in the study. Exemplary images of fibrotic quantification are depicted in Fig. 2. Analysis of OFL and LSS using the opacity-based Pneumonia tool took an average of 611.75 ± 60.8s, analysis of MTFL is automated and required 312.61 ± 25.19s (*P* < 0.001). Inter-observer variability analysis showed very high agreement (ICC: OFL 0.91 [CI: 0.84–0.95], LSS 0.92 [CI: 0.86–0.96])^20^ for manual refinement of OFL segmentation in 24 of 45 cases.

**Fig. 3:**
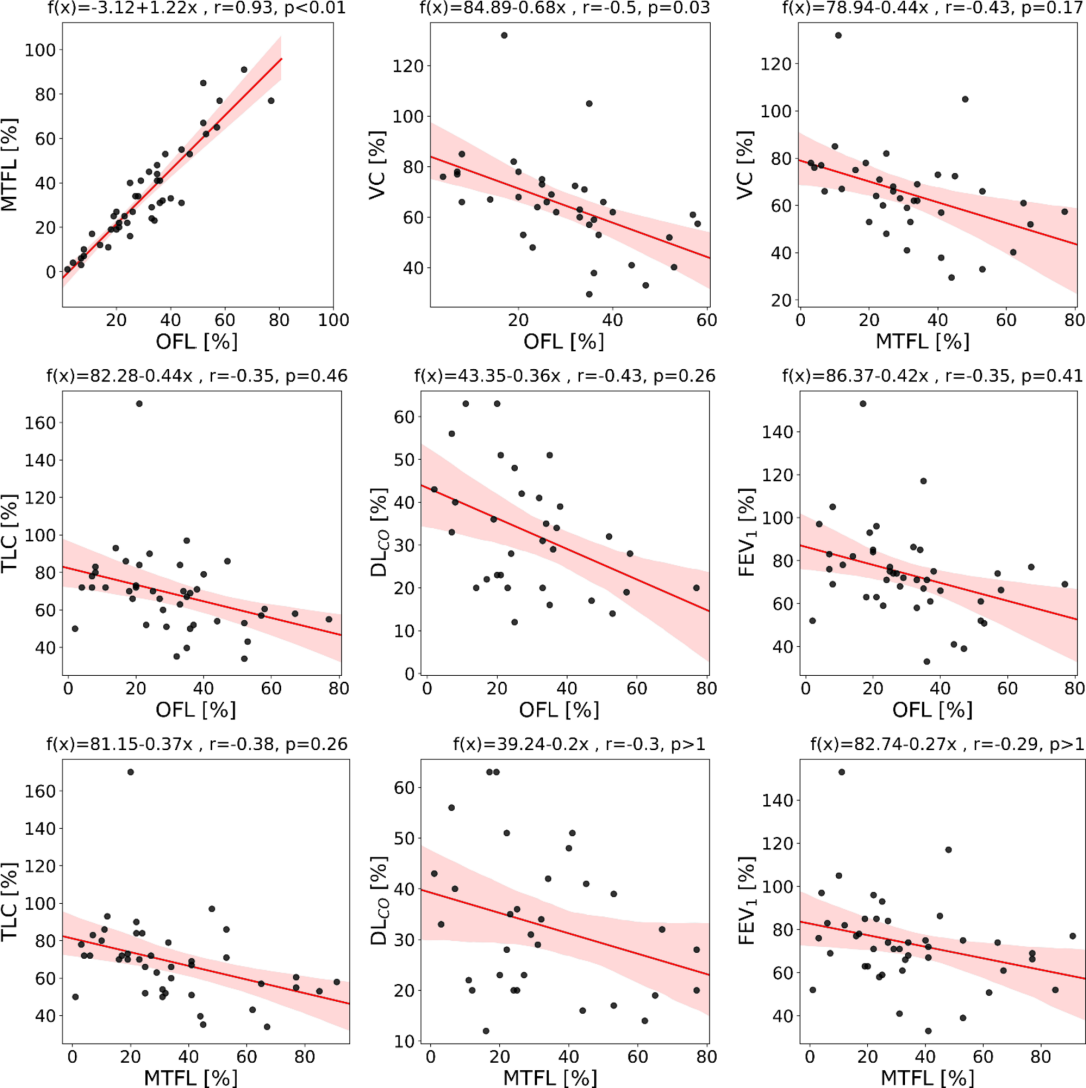
Linear regression analyses. Illustration of regression analysis between the opacity-based fibrotic lung (OFL) and the multi-texture fibrotic lung (MTFL) quantification, as well as regression analysis of OFL and MTFL with spirometry parameters with Pearson correlation coefficient (r) bigger than 0.25. 95% confidence intervals are indicated by light-colored areas. *TLC* percent predicted total lung capacity, *VC* percent predicted vital capacity and *DL*_*CO*_ diffusing capacity of the lungs for carbon monoxide.

**Fig. 4:**
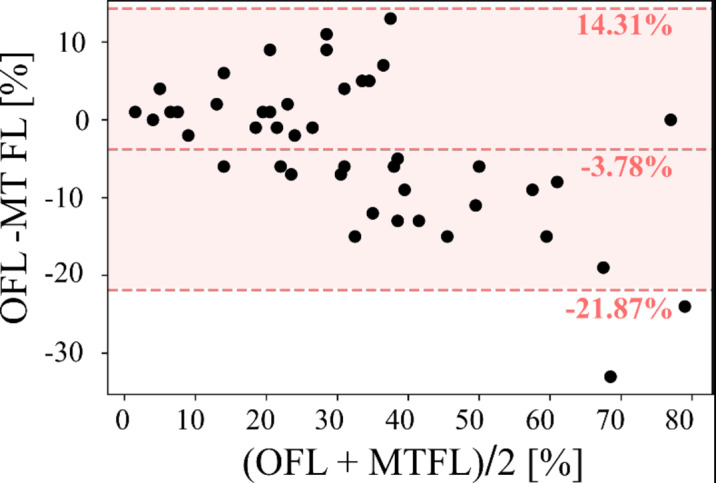
Bland-Altman analysis. The opacity-based fibrotic lung (OFL) quantifications was compared to the multi-texture fibrotic lung (MTFL) measurements.

Median with inter quartile ranges of OFL, LSS and MTFL, as well as general information on patient characteristics can be found in Table 1. The quantifications of OFL and MTFL did not differ (median and interquartile range: OFL 29% [20-38%], MTFL 31% [19-45%]; *P* = 0.44). Also, high correlation was observed between OFL and MTFL (*r* = 0.93, *p* < 0.01). Table 2 shows the results of the regression analysis between OFL, LSS and MTFL and the subsets of patients for which the spirometry parameters TLC, VC, FEV_1_, DL_CO_, P_O2_ and P_CO2_ were available. For OFL (*P* = 0.03) and LSS (*P* = 0.05) significant correlation to VC was observed. No significant correlation was found MTFL after Bonferroni correction. Figure 3 illustrates linear regression analysis of OFL and MTFL to spirometry parameters. Bland-Altman analysis (Fig. 4) reveals a bias of -3.78% and 95% limits of agreement of -21.87–14.31% with OFL being lower than MTFL.

**Table 1:** General characteristics and imaging quantifications of the patients included in the study.

Imaging quantifications		Risk factors	
OFL	29% [20–38]	Active smoker	21 (47%)
LSS	10 [7 - 12]	Stress	7 (16%)
MTFL	31% [19–45]	Allergies	10 (22%)
Basic attributes		Symptoms	
Age	76 years [67–81]	Coughing	16 (36%)
Male	37 (82%)	Dyspnea	37 (82%)
Spirometry parameters		Comorbidities	
TLC (*N* = 42)	69.5% [54.2–78.8]	Hypertonia	28 (62%)
VC (*N* = 36)	65.0% [56.0-73.5]	Diabetes	10 (22%)
FEV_1_ (*N* = 43)	72.0% [63.0-82.5]	CHD	17 (38%)
DL_CO_ (*N* = 32)	31.5% [20.0-41.2]	Hyperlipidemia	14 (31%)
P_O2_ (*N* = 45)	67.0 mmHg [59.1–74.7]		
P_CO2_ (*N* = 45)	36.0 mmHg [34.0-37.8]		

For the opacity-based fibrotic lung (OFL) quantification and the lung severity score (LSS), as well as for the multi-texture fibrotic lung (MTFL) quantification and other interval scaled measurements median and inter quartile ranges are given. The absolute number of patients (N) is given, for which the spirometry parameters were available. For risk factors, symptoms and comorbidities N is given. *CHD* coronary heart disease, *TLC* percent predicted total lung capacity, *VC* percent predicted vital capacity, *FEV*_*1*_ percent predicted forced expiratory volume in 1 s, *DL*_*CO*_ diffusing capacity of the lungs for carbon monoxide, *PO2* partial pressure of oxygen, *PCO2* partial pressure of carbon dioxide.

**Table 2:** Linear regression analyses.

Versus	OFL			LSS			MTFL		
	Lin. function	*r*	*P*	Lin. function	*r*	*P*	Lin. function	*r*	*P*
TLC (*N* = 42)	82.28-0.44x	− 0.35	0.46	88.0-1.99x	− 0.34	0.54	81.15-0.37x	− 0.38	0.26
VC (*N* = 36)	84.89-0.68x	− 0.5	**0.03**	93.46-3.02x	− 0.49	**0.05**	78.94-0.44x	− 0.43	0.17
FEV_1_ (*N* = 43)	86.37-0.42x	− 0.35	0.41	91.13-1.82x	− 0.33	0.56	82.74-0.27x	− 0.29	1
DL_CO_ (*N* = 32)	43.35-0.36x	− 0.43	0.26	48.68-1.69x	− 0.43	0.26	39.24-0.2x	− 0.3	1
P_O2_ (*N* = 45)	64.73 + 0.09x	0.1	1	61.91 + 0.59x	0.14	1	65.38 + 0.06x	0.09	1
P_CO2_ (*N* = 45)	34.33 + 0.06x	0.17	1	32.57 + 0.36x	0.23	1	35.55 + 0.02x	0.06	1

The opacity-based fibrotic lung (OFL) quantification and the lung severity score (LSS), as well as for the multi-texture fibrotic lung (MTFL) quantification were compared with the spirometry parameters percent predicted total lung capacity (TLC), percent predicted vital capacity (VC), percent predicted forced expiratory volume in 1 s (FEV_1_), diffusing capacity of the lungs for carbon monoxide (DL_CO_), partial pressure of oxygen (P_O2_) and carbon dioxide (P_CO2_) in a subset of N patients, for whom the parameters were available. The linear function, Pearson correlation coefficient (r) and p-value are given for each regression analysis. P–values were corrected for multiple comparison of 19 regression analyses conducted by Bonferroni method. Significant values are in bold.

As shown in Table 3, only the image-based markers (OFL *P* = 0.03, LSS *P* < 0.01 and MTFL *P* = 0.02) showed significant association with the survival of the patients in multivariable CPH analyses with age, sex and TLC. The CPH model, which solely included patients with reported VC, resulted in neither the image-based markers (OFL *P* = 1, LSS *P* = 0.73, and MTFL *P* = 1) nor sex, age, or VC showing a significant association with patient hazard. In univariable CPH analysis, VC (0.97 [0.93-1.00], *P* = 0.07) showed a greater, yet also non-significant, association with patient hazard compared to TLC (1.00 [0.97-1.02], *P* = 0.82).

**Table 3:** Multivariable cox-proportional-hazards analysis.

	OFL		LSS		MTFL		OFL		LSS		MTFL	
	Hazard ratio	*P*	Hazard ratio	*P*	Hazard ratio	*P*	Hazard ratio	*P*	Hazard ratio	*P*	Hazard ratio	*P*
Image-based	1.03 [1.01–1.06]	**0.03**	1.25 [1.09–1.44]	**< 0.01**	1.03 [1.01–1.05]	**0.02**	1.01 [0.97–1.05]	1	1.15 [0.91–1.44]	0.73	1.01 [0.98–1.04]	1
Sex	2.12 [0.48–9.41]	0.98	2.41 [0.54–10.69]	0.74	2.14 [0.48–9.47]	0.95	4.20 [0.54–32.79]	0.51	4.59 [0.58–36.06]	0.44	4.21 [0.54–32.87]	0.51
Age	1.06 [0.99–1.13]	0.33	1.07 [1.00-1.15]	0.17	1.05 [0.98–1.12]	0.60	1.06 [0.99–1.15]	0.33	1.07 [0.99–1.16]	0.25	1.06 [0.99–1.15]	0.34
TLC (*N* = 42)	1.00 [0.98–1.03]	1	1.01 [0.99–1.03]	1	1.01 [0.99–1.03]	1	–	–	–	–	–	–
VC (*N* = 36)	–	–	–	–	–	–	0.97 [0.93–1.01]	0.53	0.98 [0.94–1.02]	0.98	0.97 [0.93–1.01]	0.49

The prognostic value for 3-year mortality of patients with idiopathic pulmonary fibrosis was investigated in multivariable cox-proportional-hazards models including basic clinical attributes (sex, age) and the spirometry parameter total lung capacity (TLC) and in models including sex, age and the spirometry parameter vital capacity (VC). For each analysis, only the number of patients (N) were included for whom TLC or respective VC were available. The analysis was repeated for the opacity-based fibrotic lung (OFL) quantification and the lung severity (LSS), as well as for the multi-texture fibrotic lung (MTFL) quantification. The analysis was corrected for multiple comparison with Bonferroni method. Significant values are in bold.

**Fig. 5:**
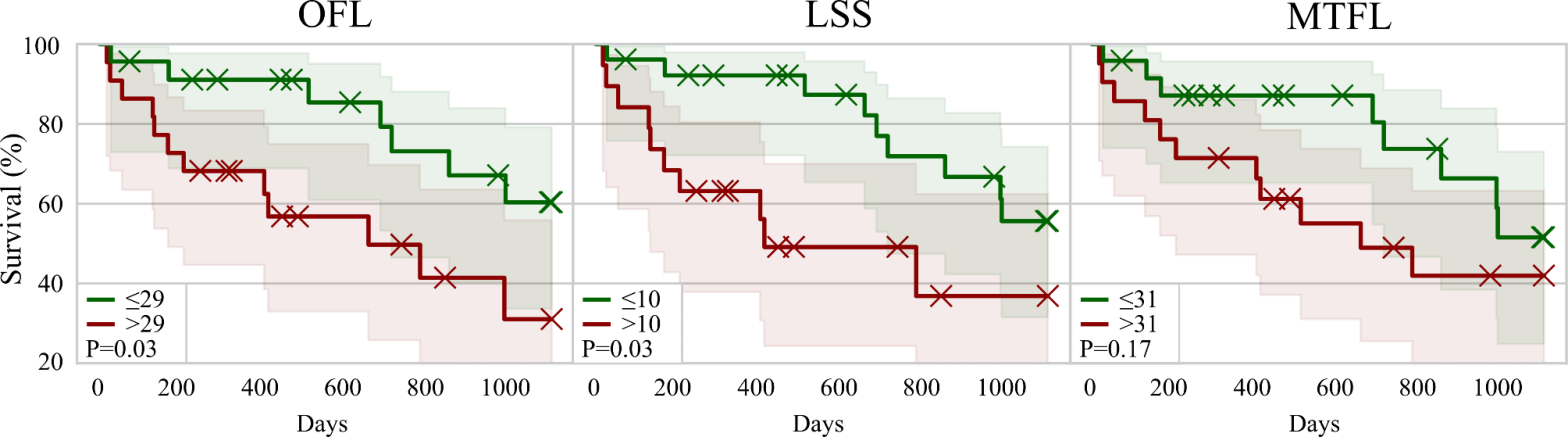
Kaplan-Meier survival plots. Patients were separated by the median of the opacity-based fibrotic lung (OFL) quantification, by the lung severity score (LSS) and by the multi-texture fibrotic lung (MTFL) quantification. Differences between groups were tested by log rank test.

Figure 5 shows the Kaplan-Meier analysis for patient groups separated by median OFL, LSS and MTFL with log rank test of significant differences. For OFL (*P* = 0.03) and LSS (*P* = 0.03) significant differences were observed but not for MTFL (*P* = 0.17).

## Discussion

In this study, an opacity-based analysis tool, initially developed for identification of inflammatory infiltrates, was compared to an established multi-texture lung analysis tool. The quantitative assessment of pulmonary fibrosis and prognosis estimation in patients with IPF was evaluated.

Employing automated pulmonary analysis for quantification of pulmonary fibrosis holds several advantages. Unlike visual approximation, which is prone to inter-observer variability and subjectivity, automated tools offer the prospect of a more objective and standardized approach. Nevertheless, visual approximation remains the current clinical standard for quantification of fibrosis.

The clinical need for affordable and simple tools to quantify fibrosis in CT imaging is paramount, especially since introduction of progressive pulmonary fibrosis as an independent fibrotic entity^21^. While specialized tools may offer more targeted analysis for certain pathologies and use cases, such as the discrimination of sub-textures of fibrotic lung, tools with more widespread and general application to multiple use cases might be preferable, particularly in resource-constrained environments.

Initially designed to quantify inflammatory infiltrates, specifically pneumonia, the opacity-based Pneumonia tool might have potential to the assessment of various pulmonary abnormalities characterized by reduced aeration or ‘consolidations’.

The results of the current study show that OFL quantification of this tool did not significantly differ and showed high correlation in linear regression analysis to MTFL measured by the sophisticated CALIPER lung texture analysis tool. Bland-Altman analysis reveal a small systematic difference of OFL to MTFL of -3.78%, with OFL being lower. As already indicated, the CALIPER tool was trained explicitly for the detection of fibrotic subtypes, and the Pneumonia tool was originally conceptualized to quantify the extent of pulmonary infectious disease. Therefore, slight differences between both tools were to be expected. Considering that results from visual approximation of fibrotic extent data severely differ from automated approaches^22^, as recently reported, smaller differences between the two automatic approaches are most likely acceptable for clinical purposes.

For quantification of the amount of MTFL, the combined quantification of lung with ground glass, reticulation and honeycombing features was calculated to allow for comparability with the quantified OFL, which represents an opacity-based, binary differentiation of diseased and to non-diseased lung. Recently it has been shown that the combination of fibrotic features in a single score appears to have superior correlation with clinical features such as lung function and outcome^23^, thus disadvantages are not to be expected in outcome prediction when solely employing binary opacity detection by the Pneumonia tool without discrimination of various fibrotic features. Nevertheless, fibrotic subtype classification and estimation of inflammation are easier when using a tool that allows for discrimination of fibrotic features^24^. Inter-reader reproducibility was only assessed for the Pneumonia tool, as the CALIPER multi-texture lung analysis tool does not enable modification of segmentations. Reproducibility of the Pneumonia tool for opacity detection was higher compared to recently reported results of visual assessment^22^.

However, manual interaction resulted in opacity detection by the Pneumonia tool taking considerably longer. Although not examined in the current study, differences in duration of analysis will be even longer when compared to the visual approximation, as data extraction, computation time and manual corrections are not necessary when visually approximating the extent of fibrosis. A faster analysis could improve the clinical applicability in the future.

No substantial difference in association of OFL and MTFL with the prognosis of 42 IPF patients was observed in multivariable CPH models along age, sex and TLC. When including VC instead of TLC, no parameter was significantly associated with the hazard of the 36 included patients. One might speculate that these insignificant results are due to the lower number of included patients with available VC, due to collinearities between VC and the imaging parameters, or due to FVC being more tightly linked to outcomes than TLC^25^. Furthermore, Kaplan-Meier analysis indicate association of OFL to patient survival that could not be seen for MTFL.

Previously works demonstrated correlation between densitometric-based analysis in pulmonary fibrosis and forced VC / DL_CO_^26^. The current results add results on correlation amount of opacified lung measured by the Pneumonia tool to VC and to MTFL determined by the CALIPER software.

The lower P-values in multivariable CPH analysis might indicate that the local assessment of opacities (i.e. the extent of pulmonary abnormalities in all lung lobes) of the LSS created by the Pneumonia tool could be beneficial for predicting patient prognosis compared to the total amount of OFL. The CALIPER lung texture analysis tool does not allow for calculation of a regional score of fibrosis severity. Overall, there are no significant differences in usability between the tools, except for the fact that the opacity-based Pneumonia tool allows for adjustments. This manual adjustments tend to increase duration of analyses, however the prospect of visual review of segmentation with possible correction leads to a better comprehensibility of results. Although the binary identification of OFL included lung tissue sections with several hyperdense fibrotic features (reticulations, ground-glass, consolidations), some partially hyperlucent alterations (such as honey-combing) were only partially detected in some cases. This resulted in manual adjustment of OFL segmentations in more than half of the cases and could explain the systematic difference revealed in Bland-Altman analysis. In the current study only IPF cases were included; currently, no data is available that indicates whether the results extends to other pulmonary fibrotic diseases.

## Conclusion

For the current cohort of IPF patients, no substantial difference in quantification of disease, correlation to spirometry parameters and association with patient prognosis was observed between the dedicated and established CALIPER multi-texture lung analysis tool and the investigated opacity-based Pneumonia tool. Although requiring manual interaction, the opacity-based Pneumonia tool could have potential for more general quantification of pulmonary abnormalities, besides the identification of inflammatory infiltrates it was developed for.

## Data Availability

The datasets used and/or analysed during the current study are available from the corresponding author on reasonable request.

## Abbreviations

IPF: Idiopathic pulmonary fibrosis
OFL: Opacity-based fibrotic lung
MTFL: Multi-texture based fibrotic lung
r: Pearson correlation coefficient
TLC: Percent predicted total lung capacity
VC: Percent predicted vital capacity
FEV1: Percent predicted forced expiratory volume in 1 s
DLCO: Diffusing capacity of the lungs for carbon monoxide
PO2: Partial pressure of oxygen
PCO2: Partial pressure of carbon dioxide
CPH: Cox-proportional-hazards
PF-ILD: Progressive fibrosis in interstitial lung
LSS: Lung severity score
ICC: Intra-class correlation coefficient
CI: 95% confidence intervals

## References

[1] Lederer, D. J. & Martinez, F. J. Idiopathic pulmonary fibrosis. *N. Engl. J. Med.* **10** (19), 1811–1823 (2018). 10.1056/NEJMra1705751 29742380

[2] Raghu, G. et al. An official ATS/ERS/JRS/ALAT statement: idiopathic pulmonary fibrosis: evidence-based guidelines for diagnosis and management. *Am. J. Respir Crit. Care Med.* **183** (6), 788–824 (2011). 21471066 10.1164/rccm.2009-040GL PMC5450933

[3] Hutchinson, J., Fogarty, A., Hubbard, R. & McKeever, T. Global incidence and mortality of idiopathic pulmonary fibrosis: a systematic review. *Eur. Respir. J.* **46** (3), 795–806 (2015). 25976683 10.1183/09031936.00185114

[4] Lamas, D. J. et al. Delayed access and survival in idiopathic pulmonary fibrosis. *Am. J. Respir Crit. Care Med.* **184** (7), 842–847 (2011). 21719755 10.1164/rccm.201104-0668OC PMC3208648

[5] Hewson, T. et al. Timing of onset of symptoms in people with idiopathic pulmonary fibrosis. *Thorax* **73** (7), 683–685 (2018). 10.1136/thoraxjnl-2017-210177 29021387

[6] Bartholmai, B. J. et al. Quantitative CT IMAGING of interstitial lung diseases. *J. Thorac. Imaging* **28**(5) (2013). 10.1097/RTI.0b013e3182a21969 PMC3850512 23966094

[7] Chen, A., Karwoski, R. A., Gierada, D. S., Bartholmai, B. J. & Koo, C. W. Quantitative CT analysis of diffuse lung disease. *Radiographics* **40**, 28–43 (2020). 31782933 10.1148/rg.2020190099

[8] Flaherty, K. R. et al. Nintedanib in progressive fibrosing interstitial lung diseases. *N. Engl. J. Med.* **381**, 1718–1727 (2019). 31566307 10.1056/NEJMoa1908681

[9] Watadani, T. et al. Interobserver variability in the CT assessment of honeycombing in the lungs. *Radiology* **266** (3), 936–944 (2013). 23220902 10.1148/radiol.12112516

[10] Maldonado, F. et al. Automated quantification of radiological patterns predicts survival in idiopathic pulmonary fibrosis. *Eur. Respir. J.* **43** (1), 204–212 (2014). 23563264 10.1183/09031936.00071812

[11] Jacob, J. et al. Automated quantitative computed tomography versus visual computed tomography scoring in idiopathic pulmonary fibrosis. *J. Thorac. Imaging* **1** (5), 304–311 (2016). 10.1097/RTI.0000000000000220 27262146

[12] Park, H. J. et al. Texture-based automated quantitative assessment of regional patterns on initial CT in patients with idiopathic pulmonary fibrosis: relationship to decline in forced vital capacity. *Am. J. Roentgenol.* **207** (5), 976–983 (2016). 27533069 10.2214/AJR.16.16054

[13] Fidler, L. & Shapera, S. Diagnostic criteria for idiopathic pulmonary fibrosis. *Lancet Respir. Med.* **6** (2), e6 (2018). 29413089 10.1016/S2213-2600(18)30020-1

[14] Chaganti, S. et al. Automated quantification of CT patterns associated with COVID-19 from chest CT.* Radiol. Artif. Intell.*** 2**(4), e200048 (2020). 10.1148/ryai.2020200048 PMC7392373 33928255

[15] Bernheim, A. et al. Chest CT findings in coronavirus disease-19 (COVID-19): relationship to duration of infection. *Radiology* **295**, 200463 (2020). 32077789 10.1148/radiol.2020200463 PMC7233369

[16] Zavaletta, V. A., Bartholmai, B. J. & Robb, R. A. High resolution multidetector CT-Aided tissue analysis and quantification of lung fibrosis. *Acad. Radiol.* **14** (7), 772–787 (2007). 17574128 10.1016/j.acra.2007.03.009 PMC2701291

[17] Pedregosa, F. et al. Scikit-learn: machine learning in Python. *J. Mach. Learn. Res.* **12**, 2825–2830 (2011).

[18] Virtanen, P. et al. SciPy 1.0: fundamental algorithms for scientific computing in Python. *Nat. Methods*. **17** (3), 261–272 (2020). 32015543 10.1038/s41592-019-0686-2 PMC7056644

[19] Davidson-Pilon, C. Lifelines: survival analysis in Python. *J. Open. Source Softw.* **4** (40), 1317 (2019).

[20] Koo, T. K. & Li, M. Y. A Guideline of selecting and reporting intraclass correlation coefficients for reliability research. *J. Chiropr. Med.* **15**, 155–163 (2016). 27330520 10.1016/j.jcm.2016.02.012 PMC4913118

[21] Cottin, V. Criteria for progressive pulmonary fibrosis: getting the horse ready for the cart. *Am. J. Respir. Crit. Care Med.* **207**, 11–13 (2023). 36066856 10.1164/rccm.202208-1639ED PMC9952875

[22] Landini, N. et al. CT evaluation of interstitial lung disease related to systemic sclerosis: visual versus automated assessment. A systematic review. *Clin. Radiol.* **79** (3), e440–e452 (2024). 38143228 10.1016/j.crad.2023.11.022

[23] Fraser, E., St Noble, V., Hoyles, R. K., Benamore, R. & Ho, L-P. Readily accessible CT scoring method to quantify fibrosis in IPF. *BMJ Open Respir. Res.* **7**, e000584 (2020). 32527873 10.1136/bmjresp-2020-000584 PMC7292044

[24] Remy-Jardin, M. et al. Importance of ground-glass attenuation in chronic diffuse infiltrative lung disease: pathologic-CT correlation. *Radiology* **189**, 693–698 (1993). 8234692 10.1148/radiology.189.3.8234692

[25] Babalola, B. T. & Yahya, W. B. Effects of collinearity on Cox proportional hazard model with time dependent coefficients: a simulation study. *J. Biostat. Epidemiol.* **5**(2) (2019).

[26] Ash, S. Y. et al. Densitometric and local histogram based analysis of computed tomography images in patients with idiopathic pulmonary fibrosis. *Respir. Res.* **18** (1), 45 (2017). 28264721 10.1186/s12931-017-0527-8 PMC5340000

